# Locus ceruleus neurons in people with autism contain no histochemically-detectable mercury

**DOI:** 10.1007/s10534-015-9898-9

**Published:** 2015-11-27

**Authors:** Roger Pamphlett, Stephen Kum Jew

**Affiliations:** Discipline of Pathology, The University of Sydney, Brain and Mind Centre, Camperdown, NSW 2050 Australia

**Keywords:** Autism, Mercury, Locus ceruleus, Locus coeruleus, Toxicant, Heavy metal

## Abstract

Exposure to environmental mercury has been proposed to play a part in autism. Mercury is selectively taken up by the human locus ceruleus, a region of the brain that has been implicated in autism. We therefore looked for the presence of mercury in the locus ceruleus of people who had autism, using the histochemical technique of autometallography which can detect nanogram amounts of mercury in tissues. In addition, we sought evidence of damage to locus ceruleus neurons in autism by immunostaining for hyperphosphorylated tau. No mercury was found in any neurons of the locus ceruleus of 6 individuals with autism (5 male, 1 female, age range 16–48 years). Mercury was present in locus ceruleus neurons in 7 of 11 (64 %) age-matched control individuals who did not have autism, which is significantly more than in individuals with autism. No increase in numbers of locus ceruleus neurons containing hyperphosphorylated tau was detected in people with autism. In conclusion, most people with autism have not been exposed early in life to quantities of mercury large enough to be found later in adult locus ceruleus neurons. Human locus ceruleus neurons are sensitive indicators of mercury exposure, and mercury appears to remain in these neurons indefinitely, so these findings do not support the hypothesis that mercury neurotoxicity plays a role in autism.

## Introduction

Environmental toxins (i.e., toxicants) have long been suspected to play a role in the pathogenesis of autism, but there is still disagreement as to what toxicants, if any, could be involved (Lyall et al. 2014). One toxicant that has been repeatedly implicated in autism is mercury (Kern et al. 2012; Mutter et al. 2005). Although some autism studies have reported environmental exposures to mercury during early life, and measured various biomarkers of mercury exposure, no consensus as to the role of mercury in autism has been reached. One area of research in this field where data are notably lacking is in assessments of toxic metals such as mercury in the brains of people with autism.

The region of the human brain that takes up most mercury is the locus ceruleus in the pons (Pamphlett and Kum Jew 2013b), probably because these neurons innervate most of the blood vessels in the brain and so are exposed to circulating toxins (Pamphlett 2014). Once mercury enters locus ceruleus neurons it appears to remain there indefinitely (Pamphlett and Kum Jew 2015), which suggests the locus ceruleus can act as a indicator of past exposure to this toxic metal. Human exposure to mercury appears to be quite common, since 43 out of 60 (72 %) adults without neurological or psychiatric disorders have been shown to have mercury within locus ceruleus neurons (Pamphlett and Kum Jew 2013a; 2015). A decrease of noradrenaline output from locus ceruleus neurons has been postulated to underlie some of the features of autism (Martchek et al. 2006; Mehler and Purpura 2009), so it seems worthwhile to look for the presence of mercury in the locus ceruleus in people who have had autism.

A histochemical technique that can detect nanogram amounts of mercury in tissues is autometallography (Danscher and Moller-Madsen 1985), so we used this method to look for evidence of mercury in locus ceruleus neurons in people with autism compared to controls. We also looked for evidence of damage to locus ceruleus neurons in autism by staining for hyperphosphorylated tau (Braak and Del Tredici 2011).

## Methods

### Cases and controls

7 μm paraffin sections of pons containing the locus ceruleus were available from 6 individuals with autism from the Oxford Brain Bank (5 male, 1 female, mean age 27 years, age range 16–48 years). The diagnosis of autism was made during life on Autism Diagnostic Interview-Revised criteria. Causes of death were three cases of sudden unexpected death in epilepsy and one each of cystic fibrosis, glioblastoma multiforme, and water intoxication. Control stained sections of pons were available from 5 neurologically-normal controls (3 male, 2 female, age range 26–47 years) and 6 patients with amyotrophic lateral sclerosis (5 male, 1 female, age range 38–49 years) from previous studies of locus ceruleus neurons (Pamphlett and Kum Jew 2013a, 2015).

### Autometallographic staining for mercury

Sections were stained with silver nitrate autometallography, which under routine conditions stains the sulphides or selenides of mercury, silver, and bismuth (Danscher and Moller-Madsen 1985; Danscher et al. 2000). The silver-coated deposits of these metals in tissues are seen histologically as black-staining grains, and referred to as AMG^HM^ (autometallography-demonstrable heavy metals). Sections were placed in physical developer containing gum arabic, citrate buffer, hydroquinone and silver nitrate at 26 °C for 80 min in the dark, then washed in sodium thiosulphate to remove unbound silver. Sections were counterstained with mercury-free haematoxylin and viewed under bright-field microscopic illumination at 600 × magnification. A positive control section was included of mouse spinal motor neurons that contained mercury following intraperitoneal exposure to mercuric chloride (Pamphlett and Png 1998). The percentage of locus ceruleus neurons containing AMG^HM^ was calculated as previously described (Pamphlett and Kum Jew 2015).

### AT8 staining for hyperphosphorylated tau

Paraffin sections were treated with hydrogen peroxide followed by normal horse serum and incubated in the monoclonal antibody AT8 AntiHuman Phos PHF Tau pSer202/Thr 205 (Thermo MN1020) at 1:1000 for 60 min at 37 °C. Slides were incubated with Envision Dual Link HRP and visualized with 3,3 diaminobenzidine tetrahydrochloride, then counterstained with haematoxylin and viewed with bright-field microscopy.

### Ethics

Sections of post mortem paraffin-embedded pons were obtained after application to the UK Oxford Brain Bank, which with approval from a research ethics committee collects and distributes consented donated brain tissue for research. No patient consent specifically for this project was therefore applicable. The work was carried out in accordance with the ethical standards of the Human Ethics Review Committee (RPAH Zone) of the Sydney Local Health District (X14-0029) and with the Declaration of Helsinki as revised in 2000. The authors declare they have no conflicts of interest.

## Results

### Autometallography

**Fig. 1:**
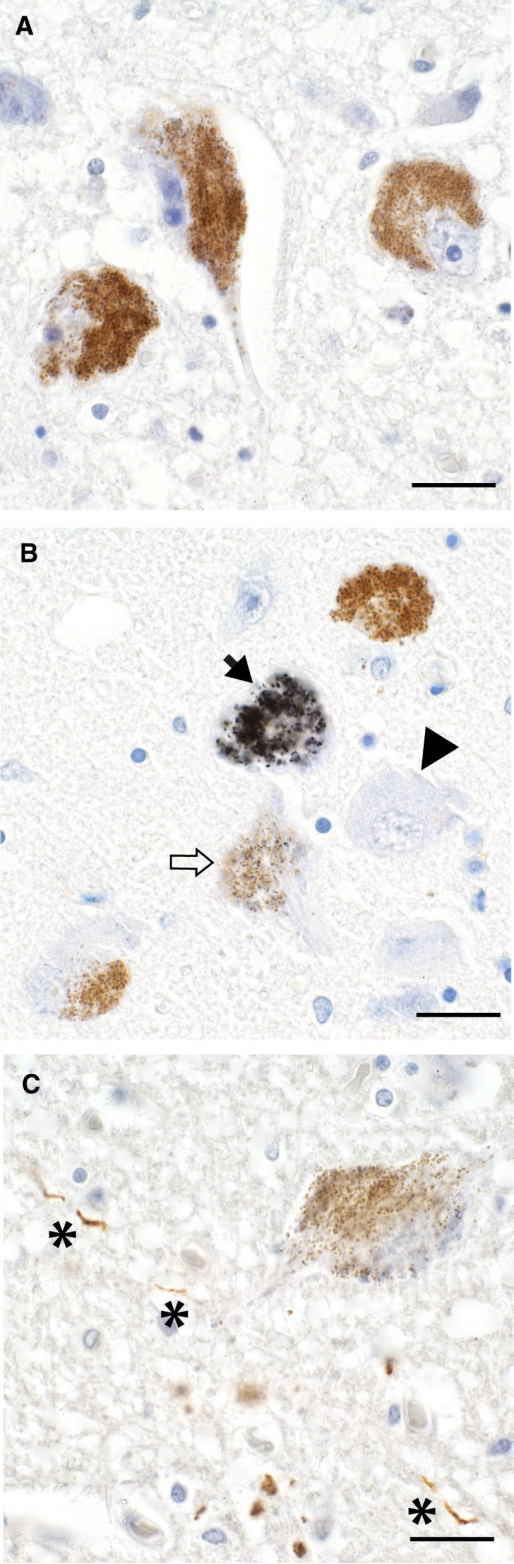
**a** Three locus ceruleus neurons in this individual with autism contain yellow–brown neuromelanin pigment, but no black grains of autometallographically-detectable mercury (AMG^HM^). **b** One locus ceruleus neuron in this patient with amyotrophic lateral sclerosis shows widespread (*closed arrow*), and one scattered (open arrow), AMG^HM^; other neurons, including a non-pigmented neuron (*arrow*-*head*) show no AMG^HM^. **a**, **b** Autometallography and hematoxylin. **c** Staining for hyperphosphorylated tau is seen in a locus ceruleus neurite (*asterisks*) in this individual with autism, but not in the cell body of an adjacent pigmented neuron. AT8 immunostaining and hematoxylin. All *bars* 20 μm

No AMG^HM^ was seen in locus ceruleus neurons of any of the six individuals with autism (Fig. 1a). In comparison with age-matched controls, using the same AMG technique, two of five neurologically-normal controls had locus ceruleus neurons containing AMG^HM^ (2 and 5 % of neurons respectively), as did five of six amyotrophic lateral sclerosis patients (4, 5, 17, 22 and 32 % of neurons respectively) (Pamphlett and Kum Jew 2013a) (Fig. 1b). The presence of AMG^HM^ in LC neurons of autism individuals was significantly less than for combined age-matched controls (Fisher’s exact two-sided p = 0.03).

### AT8 for hyperphosphorylated tau

AT8 staining was seen in one neurite in the locus ceruleus of one individual with autism (Fig. 1c), but in none of the other five individuals with autism. No cell bodies of locus ceruleus neurons of any autism individual showed AT8 staining. In comparison, using the same AT8 technique, in 12 individuals who had no neurological or psychiatric conditions (7 male, 5 female, age range 51–59 years) AT8 staining was present in locus ceruleus neurites in 3 individuals and in neuronal cell bodies in 2 individuals (Pamphlett and Kum Jew 2015).

## Discussion

No mercury was detected in locus ceruleus neurons of people with autism using a sensitive histochemical technique that can show the presence of nanogram amounts of mercury. This is despite the locus ceruleus being the region of the human brain that takes up circulating mercury most avidly, and the frequent presence of mercury in these neurons in control individuals. Furthermore, no signs of damage to the locus ceruleus was seen on hyperphosphorylated tau immunostaining, beyond that expected for age. These findings do not support the hypothesis that exposure to mercury early in life is a risk factor for autism, or that the locus ceruleus is damaged in autism.

The study has a number of limitations. (1) Numbers of autism post mortem samples available for examination were limited, so it remains possible that a subset of people with autism could have mercury in their locus ceruleus neurons. Autism is seldom a life-threatening disorder, so even brain banks that specialise in recruiting autism donors such as the one accessed here have only limited numbers of brain samples available for research. Nevertheless, statistically fewer people with autism had mercury in the locus ceruleus neurons than age-matched controls. (2) Trace amounts of mercury could have triggered an autoimmune response (Mutter et al. 2005) which destroyed the neurons that contained the mercury, so they would not have survived to be examined. There is however no evidence of a loss of locus ceruleus neuronal in autism (Martchek et al. 2006). (3) Mercury could transiently enter and then be cleared from locus ceruleus neurons (a “hit-and-run” effect). This seems unlikely, since neuromelanin binds heavy metals (Double et al. 2008) and the pigment appears already by the fifth month of gestation in locus ceruleus neurons (Foley and Baxter 1958). Mercury would therefore be expected to remain in these neurons indefinitely.

More work is needed to find out if a range of toxic element other than mercury could be present in the brains of people with autism. Multiple elements within cells can now be detected using synchrotron X-ray fluorescence microprobe analysis (Aitken et al. 2010). This technique could be used to look for a large number of toxic elements within locus ceruleus neurons, but this will depend on the availability of sufficient numbers of frozen pons samples from people with autism as well as controls.

In conclusion, our findings do not support the hypothesis that exposure to mercury is a risk factor for autism. The usual caveat of “absence of evidence is not evidence of absence” needs however to be heeded, and further efforts to analyse brain, and in particular locus ceruleus, toxins in autism should be encouraged.
